# Nutrient Composition and Flavor Profile of Crucian Carp Soup Utilizing Fish Residues through Comminution and Pressure-Conduction Treatment

**DOI:** 10.3390/foods13050800

**Published:** 2024-03-05

**Authors:** Qi Wang, Zheming Wang, Xiaoqing Yang, Xinru Fan, Jinfeng Pan, Xiuping Dong

**Affiliations:** 1 SKL of Marine Food Processing & Safety Control, National Engineering Research Center of Seafood, Collaborative Innovation Center of Seafood Deep Processing, Liaoning Province Collaborative Innovation Center for Marine Food Deep Processing, Dalian Technology Innovation Center for Chinese Prepared Food, School of Food Science and Technology, Dalian Polytechnic University, Dalian 116034, China; wangqi19990105@163.com (Q.W.); wangming19980729@163.com (Z.W.); yxq19970929@163.com (X.Y.); pjf613@163.com (J.P.); 2 College of Food Science and Engineering, Dalian Ocean University, Dalian 116023, China; fanxinru@dlou.edu.cn; 3 Academy of Food Interdisciplinary Science, Dalian Polytechnic University, Dalian 116034, China

**Keywords:** crucian carp, fish residue, comminution, pressure-conduction treatment, volatile compounds

## Abstract

In conventional fish soup processing, valuable aquatic resources like fish skins, bones, and scales are often squandered. This study was aimed at investigating if comminution combined with pressure-conduction treatment has the potential to enhance the reutilization of cooking residues. The different blending ratios of original soup (OS), made from the initial cooking of fish, and residue soup (RS), produced from processed leftover fish parts, were alternatively investigated to satisfy the new product development. Comminution combined with pressure-conduction treatment significantly increased the nutrient contents of calcium, soluble proteins and total solids in crucian carp soup (*p* < 0.05). With the increase in RS ratio, the decomposition of inosine monophosphate (IMP) and free amino acids was accelerated, but the accumulation of aromatic compounds was promoted simultaneously. In addition, the Maillard reaction may lead to a reduction in aldehydes, causing a diminution in the characteristic flavor of fish soup, while the formation of 1-octen-3-ol can enhance the earthiness of the fish soup. The electronic tongue test results and the sensory results showed that the blend ratio of OS and RS at 7:3 had a more significant umami and fish aroma (*p* < 0.05). Under this condition, the mixed soup has better nutritional values and flavor characteristics.

## 1. Introduction

Crucian carp, a common commercial species in Chinese aquaculture, has been traditionally consumed in the form of fish soup, which is thought to have therapeutic benefits for postpartum women by invigorating the spleen and qi, as well as aiding digestion and nourishment [1]. The soup is rich in water-soluble nutrients like collagen, free amino acids, and free fatty acids. These nutrients form micro-/nano-sized colloidal particles (MNCPs) through emulsification or the Maillard reaction, enhancing digestion and absorption in the human gastrointestinal tract. According to the previous studies, the by-product from industrialized aquatic product processing usually accounts for 30% to 70% of the total output [2]. Unfortunately, the low utilization of these by-products leads to more inefficient resource utilization and substantial environmental management costs. Traditional methods of cooking fish soup often result in the wastage of valuable components such as fish muscle, head, and bones and the loss of nutrients such as proteins, calcium, and phosphorus, which leads to the problem of the fish being loose in texture and inedible. Hence, novel strategies to enhance the reutilization of cooking residues are warranted.

Previous research studies on soup utilization mainly focused on screening heat treatment methods [3], optimizing boiling conditions [3], and exploring enzymatic hydrolysis technology [4]. Recently, researchers have had significant interest in the reuse of by-products with physical technologies. Bruno et al. [5] pretreated *Labeo rohita* heads with ultrasound pretreatment to enhance the content of polyunsaturated fatty acids in oil. Shin et al. [6] found that supercritical carbon dioxide is effective for obtaining neutral phospholipids. Compared with previously established chemical approaches, physical processing technologies can better retain the original nutrition, flavor, and safety of food while also having less environmental impact and being more readily accepted by consumers [5,6]. Compared with normal-pressure cooking, high-pressure treatment can effectively shorten the cooking time of soup, promote more nutrient migration, and improve the sensory quality of fish soup [7]. Therefore, our study adopts comminution combined with pressure-conduction cooking to enhance the utilization rate of cooking residues and release their deeper nutrients.

To the best of our knowledge, few studies have focused on the incorporation of fish residues into crucian carp soup. The main challenge is to overcome the potential impact of the mixing of fish residue on the taste, flavor, and sensory aspects of fish soup. Fish residues are a good source of bioactive compounds, including amino acids, proteins, collagen, polyunsaturated fatty acids, and vitamins [8]. Thus, to enhance soup-making techniques, maximizing raw material utilization and improving product nutrition while considering the influence of mixed residue soup (RS) ratios on flavor becomes imperative. In the realm of detecting and identifying aquatic flavor compounds, a combined approach utilizing various testing techniques has been frequently employed [2,9]. The most common methods for flavor detection are sensory evaluation and instrument analysis. Due to the high precision and sensitivity of Gas Chromatography-Mass Spectrometry (GC-MS), it has been widely applied for the qualitative and quantitative evaluation of volatile compounds in fish products.

Based on these backgrounds, this study proposes the modification of fish residue by efficient comminution combined with pressure-conduction cooking. The innovation of this study lies in the use of suitable processing techniques for fish residue to improve resource utilization and facilitate nutrient transfer from the broth. In addition, the effect of different blend ratios of crucian carp original soup (OS) and RS on the flavor and nutrient composition of the products were investigated, and the most suitable blend ratio were finally screened. The information gained from this study will provide useful references and guidance for improving the resource efficiency and environmental sustainability of aquatic product processing.

## 2. Materials and Methods

### 2.1. Materials

Crucian carp (each weight 500 ± 50 g, *n* = 9) was purchased from a local market (Qianhe Market, Dalian, China). Organic solvents (including analytical and high-performance liquid chromatography grade) were purchased from Aladdin Reagent Co., Ltd. (Shanghai, China).

### 2.2. Sample Preparation

Alive crucian carp (*n* = 3 for each group) were slaughtered, scaled, gutted, cleaned, and cut into cubes (each width: 8 ± 2 cm), respectively. To remove the muddy flavor of the soup, 0.4% (*w*/*w*) vinegar and 3% (*w*/*w*) sodium chloride were added, then stored at 25 °C for 1 h, followed by air-drying at room temperature for 2 min. Prepared fish cubes were mixed with 35 g of corn oil, grilled at 200 °C for 1 min in a frying pan, and then filtrated with kitchen paper for 2 min. The electric pressure-cooking saucepan (MY-YL50Easy203, Midea Consumer Electric Mfg Co., Ltd., Foshan, China) was employed for the processing of fish soup, as follows: the weight ratio of grilled fish and boiling water was 1: 2, and pressure was maintained at 70 kPa for 1 h.

All the boiling liquid was filtered through a 120-mesh sieve (125 μm) and left for 2 min. The filtrate was obtained as an OS. The sediment was regarded as crucian carp residue, which was further processed into RS. The preparation was as follows: Residues were pulverized for 1 min by a meat grinder (QSJ-D03Q1, Bear, Foshan, China). The crushed residues were placed in an electric pressure cooker with twice the weight of boiling water and treated at 70 kPa for 1 h. After boiling, centrifugation (4000 r/min, 15 min, 25 °C) (CAX-571, TOMY, Tokyo, Japan) was performed, and the supernatant was RS. OS and RS were mixed according to different volume ratios (10:0, 7:3, 5:5, 3:7, 0:10) to prepare mixed soup (MS) samples (marked as MS-10:0, MS-7:3, MS-5:5, MS-3:7, MS-0:10, respectively). Mixing method: Use a stirring rod to stir each group of samples clockwise for 30 s. The samples were stored at 4 °C and used within one week.

### 2.3. Total Solids Analysis

The weighing bottle was dried to obtain constant weight and was regarded as W_1_. The total weight of the 5 g sample and the aforehand dried weighing bottle were measured as W_2_ after heating at 105 °C until constant weight [10]. The results were expressed as the following equation:

$$\mathrm{Total}\;\mathrm{Solids}\;\mathrm{Analysis}\;(\%)=\frac{W_{2}-W_{1}}{5}\times 100$$

### 2.4. Soluble Proteins Analysis

The content of soluble proteins was measured using the Folin–Ciocalteu reagent described by Lowry et al. [11]. The absorbance of the mixture was measured at the absorbance of 540 nm. Calibration was achieved with a BSA standard solution (2, 4, 6, 8, and 10 mg/100 mL). 

### 2.5. Proximate Analysis

Proximate compositions were determined using the standard methods from the Association of Official Analytical Chemists (AOAC). Total proteins were determined by the AOAC Methods 988.05 [12] with an automatic Kjeldahl azotometer (A300, Membra Pure GmbH, Munich, Germany). Total lipids were determined by the AOAC Methods 963.15 [12] with a fat tester (SZF-06A, Shanghai XinJia Electronics Company, Shanghai, China). The phenol-sulfuric acid method was used to determine total sugars in samples based on the method of GB/T 9695.31 [13]. All analyses were performed in triplicate.

### 2.6. Calcium Content Analysis

Calcium contents were determined after mineralization with nitric acid in closed vessels by a microwave system (Ethos 1600 Milestone S. r. l., Sorisole, Italy) using an inductively coupled plasma optical emission spectrometer (ICP-OES, ICAP PRO Series, Thermo Fisher Scientific Inc., Waltham, MA, USA). Refer to the Manuelian et al. [14] method with slight modification. Operating conditions of the ICP-OES were plasma power of 1150 W, plasma flow of 0.5 L/min, auxiliary flow of 0.5 L/min, nebulizer flow of 12.5 L/min, and integration time of 30 s. 

### 2.7. Microstructure Measurements

The sample for laser scanning confocal microscopy (LSCM) observation was prepared as previously reported with some modifications [15,16]. The soup sample (100 µL) was stained with 10 µL Nile red (42 µg/mL in acetone) to visualize oil droplets and 5 µL Fluorescein isothiocyanate (FITC) (0.02 g/mL in ethyl alcohol) to stain proteins. Confocal images were performed on an LSCM (Andor IQ 3.2) with a 10/100× oil-immersion objective lens (laser combination: 488; 561 nm).

### 2.8. Sensory Analysis

The sensory panel was chosen in accordance with standards PN EN ISO 8586 [17], following a screening process for sensory abilities and sensitivities. All sensory panelists (*n* = 15) were previously trained in food sensory assessment and all gave informed consent to participate in the study. The appropriate protocols for protecting the rights and privacy of all participants were utilized during the execution of the research. No coercion to participate, full disclosure of study requirements and risks, written or verbal consent of participants, no release of participant data without their knowledge, ability to withdraw from the study at any time.

The sample was randomly sorted and scored by the double-blind selection method. The sample (20 mL) was reheated in a tasting cup in a microwave oven (X3-233A, Midea, Foshan, China) at 900 W for 1 min. Each panelist was provided with purified water to clear the palate between samples. The quality of crucian carp soup was evaluated within 5 min according to its color, homogeneity, fishy odor, umami, taste, and texture indexes. The scoring standards are shown in Table S1. This experimental material is a food material purchased in the market and can be safely consumed by consumers without providing ethical proof. Sensory analysis samples are to be used only on the day of sample preparation.

### 2.9. Free Amino Acid Analysis

Refer to the slightly modified method of Tanimoto et al. [18]. Briefly, 4 mL of sample was added with 1 mL of sulfosalicylic acid (10%) and refrigerated for 60 min. After centrifugation (14,500 rpm for 15 min at 4 °C), the supernatant was filtered through a 0.22 µm membrane. The volume of 20 µL for each sample was injected into the amino acid analyzer (Membra Pure A300, Bodenheim, Germany). The cationic separation column (4.6 mm × 60 mm, 3 µm) and reaction column (4.6 mm × 40 mm) were connected to the analyzer. The details of the test conditions were as follows: The mobile phases were lithium salt system buffers with a 0.25 mL/min flow rate, 70 °C column temperature, and 115 °C reactor temperature; the flow rate of the derivatizing reagent (a mass fraction of 2% ninhydrin) was 0.18 mL/min.

### 2.10. 5′-Nucleotides Analysis

Refer to the method of Qiu et al. [19]. Briefly, 5 mL of sample was added into 10 mL 5% (*v*/*v*) perchloric acid (PCA) at 4 °C, 6000× *g* for 10 min, and the supernatant was obtained, repeated twice. Then, the total supernatant was neutralized with 6 mol/L KOH to adjust pH value at 6.75. The neutral supernatant was placed in a liquid phase vial after passing through a 0.45 µm water filtration membrane for inspection. The 10 µL extracted solution was injected into a high-performance liquid chromatography (HPLC) device (LC-20A, Shimadzu, Japan) to analyze the 5′-Nucleotides with isocratic elution. Liquid chromatography conditions: equipped with a C18 column (250 mm × 4.6 mm, 5 µm, Agilent, Santa Clara, CA, USA), column temperature (30 °C), 50 mmol/L pH 6.5 ammonium acetate solution used as mobile phase. The UV detector was detected at 254 nm. Calibration was performed using nucleotide standards (0, 0.05, 0.1, 0.2, 0.3, 0.5 mg/mL).

### 2.11. Electronic Nose Detection

The electronic nose system (PEN3, Win Muster Airsense Analytics GmbH, Schwerin, Germany) was utilized to estimate the odor characteristics (Table S2) of crucian carp soup. The 5 mL of sample was taken into a headspace sampling bottle for electronic nose detection, which was immediately sealed with parafilm and incubated at room temperature (25 °C) for 30 min. Each sample was measured in parallel three times. The measured data 57–60 s after the response values were stabilized and analyzed using the built-in Win muster software (Version 1.6.2). 

### 2.12. Electronic Tongue Detection

The taste characteristics of the samples were analyzed using an electronic tongue analyzer (TS-5000Z, Insent, Tokyo, Japan), which includes umami, saltiness, sourness, bitterness, sweetness, astringency, Aftertaste-A, Aftertaste-B, and richness. The volume of 20 mL sample was diluted to100 mL with distilled water, and then transferred to the electronic tongue container waiting for the recovery of room temperature (25 °C). The response value at 120 s was selected for analysis. The washing time was 10 s between each test.

### 2.13. Volatile Compounds Analysis

Refer to the method of Ke [20]. Slightly modified. A total of 5 mL of the sample and 10 µL of cyclohexanone (100 mg/L internal standard) were placed in a headspace vial (20 mL). The volatile compounds were extracted with SPME needles having DVB/CAR/PDMS fibers (50/30 µm, Supelco, Bellefonte, PA, USA) at 50 °C for 40 min. The capillary column was a HP-5MS (30 m × 0.25 mm × 0.25 µm, Agilent, Santa Clara, CA, USA); carrier gas was helium and the flow rate was 20 mL/min. The temperature of the GC oven was kept at the initial temperature of 40 °C for 5 min, then increased to 180 °C at a rate of 4 °C/min and kept isothermal for 0 min. Finally, the column temperature was increased to 220 °C at a rate of 10 °C/min and kept for 5 min. The injection temperature was set to 250 °C. The MS conditions were as follows: the scanning range was 30–500 *m*/*z* and the ionization voltage was 70 EV; detector interface temperature was 250 °C; ion source temperature was 230 °C. For identifying volatiles, mass spectrums of volatile compounds acquired via GC-MS were compared to standard spectrums searched from NIST14 mass spectral library [21]. Compounds with a matching degree greater than or equal to 700 were selected, and miscellaneous peaks were screened. 

Determination of the key flavor compounds: with a relative odor activity value (ROAV) method of Dong et al. [22]. The greatest contribution for the total flavor was defined as ROAV_stan_, which was equal to 100. The ROAV values for other volatile components were calculated by:

$${\mathrm{ROAV}}_{i}\approx \frac{C_{\mathrm{ri}}}{C_{\mathrm{rstan}}}\times \frac{T_{\mathrm{stan}}}{T_{i}}\times 100$$

C_ri_ and T_i_ were the relative content and sensory threshold of volatile components, respectively. C_rstan_ and T_stan_ represented the relative content and sensory threshold of total flavor components within samples. Threshold values were obtained from the references [21,22]. 

### 2.14. Statistical Analysis

All the above experiments were repeated three times. For one-way analysis of variance, the Statistical Package for the Social Sciences (SPSS Inc., Chicago, IL, USA) was used (ANOVA) and further employed LSD and Duncan methods for post hoc multiple comparisons. The explanation of *p* < 0.05 was expressed as the test criterion of significance. Origin 2022 software (Origin Lab Corporation, Northampton, MA, USA) was used to plot the findings. All sensory data were collected using Sense Whisper (www.sensewhisper.com, accessed on 20 July 2022). Data analysis with PLS-DA was performed using MetaboAnalyst 6.0 (https://www.metaboanalyst.ca/, accessed on 28 December 2023).

## 3. Results and Discussion

### 3.1. Characterization of Residue Soup

The chemical compositions of OS and RS are compared in Table 1. The results showed that the nutrient dissolution of RS was better than that of OS. Especially, the calcium content of RS was 532.98 mg/L, more than 4 times that of OS. This was due to the fact that calcium mainly existed in the form of hydroxyapatite, which had low solubility [23]. Meanwhile, traditional high-pressure boiling had limited ability to promote the migration of calcium ions. This indicated that comminution could promote the dissolution of calcium ions. The soluble protein concentration of RS reached 82.36 mg/100 mL, which was six times higher than that of OS. It was demonstrated that the dispersion of raw materials was further promoted by mechanical action, resulting in a decrease in particle size and the degree of dispersion of soluble and insoluble substances in the fish soup. Soluble protein refers to the protein soluble in water in the state of small molecules, which is one of the essential indicators to measure the quality of the soup [10]. 

### 3.2. Composition and Structure in MNCPs 

**Figure 1:**
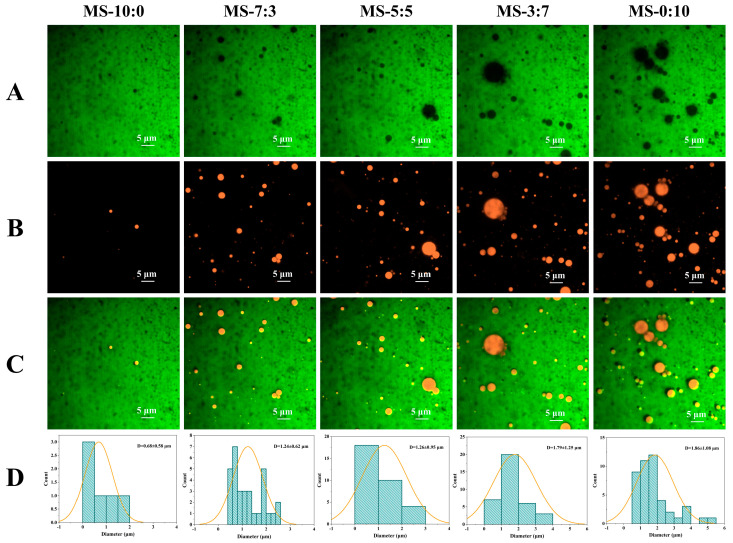
Typical CLSM images of crucian carp soup at different blend ratios. Scale bar 5 μm. (**A**) Images of protein and the polysaccharide were dyed with FITC. (**B**) Images of triglycerides were dyed with Nile Red. (**C**) Images of the overlay of the first two images. (**D**) Images of the average particle size. MS-10:0: OS and RS were mixed according to 10:0 (*v*/*v*); MS-7:3: OS and RS were mixed according to 7:3 (*v*/*v*); MS-5:5: OS and RS were mixed according to 5:5 (*v*/*v*); MS-3:7: OS and RS were mixed according to 3:7 (*v*/*v*); MS-0:10: OS and RS were mixed according to 0:10 (*v*/*v*).

In this study, the protein and the polysaccharide were labeled with the water-soluble fluorescent dye FITC (Figure 1A). Triglycerides were labeled with the lipid-soluble fluorescent dye Nile Red (Figure 1B). Previous studies found that triglycerides in MNCPs could be observed by Nile red fluorescent dye in broth [24] and big eye tuna head soup [25]. The results testified that the contents of various ingredients in soup change with different compound proportions. The OS (MS-10:0) had few particles. As the RS proportion increased, more nutrients migrated into the soups, accumulating the formation and quantity of spherical particles of different sizes in the fish soups. Measure particle length using Image J software (Version 1.52), (National Institutes of Health, Bethesda, MD, USA) and calculate the average particle size using Origin 2022. We found that the MNCPs of MS-7:3 formed stable and regular spheres with much smaller particle sizes. Overall, comminution and pressure-conduction cooking promoted the formation of MNCPs in crucian carp soup.

### 3.3. Sensory Characteristics of Crucian Carp Soup

**Figure 2:**
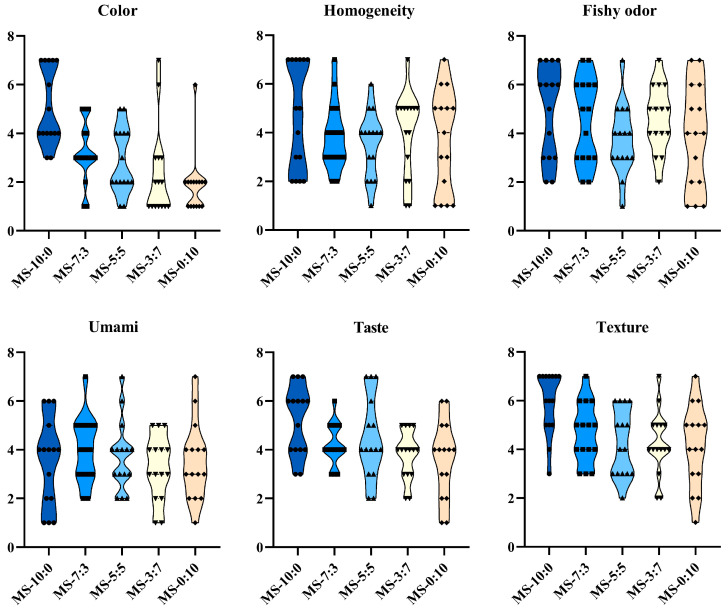
The score distribution of six sensory attributes of different blend ratios of crucian carp soup in descriptive sensory evaluation.

Sensory properties play a vital role in the development and acceptance of food products. The sensory evaluation of the crucian carp soup was based on six sensory attributes: color, homogeneity, fishy odor, umami, taste, and texture. In Figure 2 and Table S3, MS-7:3 had a significantly higher umami score than the other comparison groups (*p* < 0.05), as well as MS-10:0, which presented significantly higher scores in color, taste, and texture (*p* < 0.05), whereas MS-10:0 had a severe fishy odor synchronously. Combining the six attributes mentioned above, a comprehensive evaluation of the sensory results of crucian carp soup was conducted. The significant differences in sensory scores between groups were found to be mainly reflected in odor and taste. To further identify and analyze the causes, volatile and non-volatile compounds of the crucian carp soup were analyzed.

### 3.4. Content of Free Amino Acids in Crucian Carp Soup

**Figure 3:**
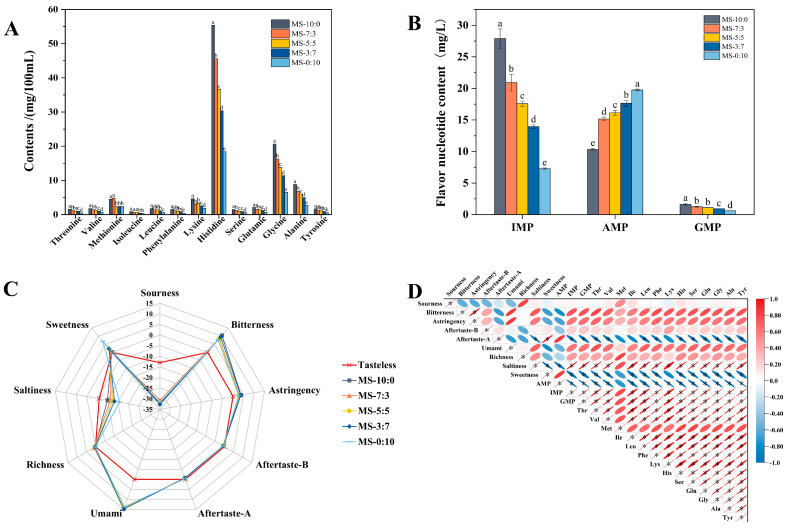
Content of free amino acids (**A**), flavor nucleotide (**B**) in crucian carp soup. Taste radar map under different blend ratios of crucian carp soup (**C**). Correlation heat map of taste profile, free amino acids, and flavor nucleotide in crucian carp soup (**D**). Different letters indicated significant difference within groups (*p* < 0.05), * indicated *p* < 0.05.

Amino acids, as flavor precursor substances, react with carbonyl compounds in the Maillard reaction to form the color and flavor of the final product. As shown in Figure 3A and Table S4, MS-10:0 had the highest total amino acid content, up to 106.53 mg/100 mL, and the content of all types of free amino acids in this group was significantly higher than that of other comparison groups (*p* < 0.05), which may indicate more affluent taste properties. Glutamic acid, glycine, and alanine constituted the umami taste, which was one of the basic tastes used in aquatic food sensory testing [26]. The high content of free amino acids and essential amino acids in crucian carp soup was histidine (55.4 mg/100 mL), which has various physicochemical functions, such as antioxidant activity, immunomodulatory activity, and anti-inflammatory activity [26]. With the increase in RS addition, the mass concentration of various free amino acids decreased, and the taste characteristics were weakened. This might be attributed to the loose state of the muscle tissue after comminution, which reabsorbed some of the amino acids in the soup. On the other hand, amino acids may be involved in the Maillard reaction and may undergo degradation to produce inosinic acid and various volatile flavor compounds as well [27].

### 3.5. Flavor Nucleotide of Crucian Carp Soup 

The taste-presenting nucleotides in crucian carp soup included inosinate acid (IMP), adenosine acid (AMP), and guanosine acid (GMP), which collectively are known as 5′-nucleotides produced through the adenosine triphosphate (ATP) degradation pathway [28]. The highest content of IMP was found in MS-10:0, reaching 27.9 mg/L (Figure 3B), demonstrating the high temperature and high-pressure treatment may facilitate the degradation of ATP to IMP. The highest content of AMP was found in MS-0:10 at 19.76 mg/L, significantly higher than the other four groups (*p* < 0.05), which may be due to the fact that comminution promoted the conversion of the ATP decomposition to AMP. The low content of GMP in the crucian carp soup was mainly due to its lower thermal stability and severe degradation under high temperature and pressure conditions [29]. Therefore, it was speculated that it did not make much contribution to the overall freshness of the crucian carp soup. IMP and glutamate synergistically interacted to enhance food freshness further [30]. Therefore, the higher concentration of 5′-IMP and glutamate contained in MS-10:0 was one of the reasons for its higher taste score in the sensory evaluation.

### 3.6. E-Tongue Analyses of Crucian Carp Soup

The e-tongue taste radar plots for different blend ratios of crucian carp soup are shown in Figure 3C, where Tasteless represents the reference solution. The Sourness, Saltiness, Aftertaste-B, and Aftertaste-A values of the crucian carp soup were all lower than those of the Tasteless point, as displayed in Figure 3C, which indicated that the crucian carp soup did not possess the above four flavor characteristics. In contrast, the other taste indicators were all valid indicators of its taste. The crucian carp soup had more noticeable bitterness and astringency values, which could be related to the fact that the crucian carp soup was richer in histidine and that the more bitter the sample, the more astringent it is. Richness refers to the enduring post-taste that emerges from the longevity of freshness within one sample. The richness values of the crucial carp soup were not substantial, with the largest richness value of MS-7:3 reaching 0.8. MS-0:10 had the minimal Umami value, but the largest sweet value reaching 6.78. These results indicated that comminution combined with pressure-conduction cooking can predominantly alter the sweet, umami, and bitter components of crucian carp soup. 

The primary taste components of crucian carp soup typically consisted of free amino acids and nucleotides, which directly or indirectly contributed to its flavor profile. Figure 3D represents the correlation analysis of the taste profile, free amino acids, and flavor nucleotides of crucian carp soup under different blend ratios between OS and RS. AMP was positively correlated with Aftertaste-A and Sweetness, and also contributed to the lower MS-0:10 sensory score. Saltiness showed significant correlations with IMP, GMP, and almost all free amino acids detected (*p* < 0.05). Umami is one of the most critical taste attributes of fish soup products. Glu, IMP, and GMP, which were highly correlated with Umami, had a more significant influence on the freshness of crucian carp soup. Met also showed a strong correlation with Richness in the MS-7:3 sample.

### 3.7. E-Nose Analyses of Crucian Carp Soup

**Figure 4:**
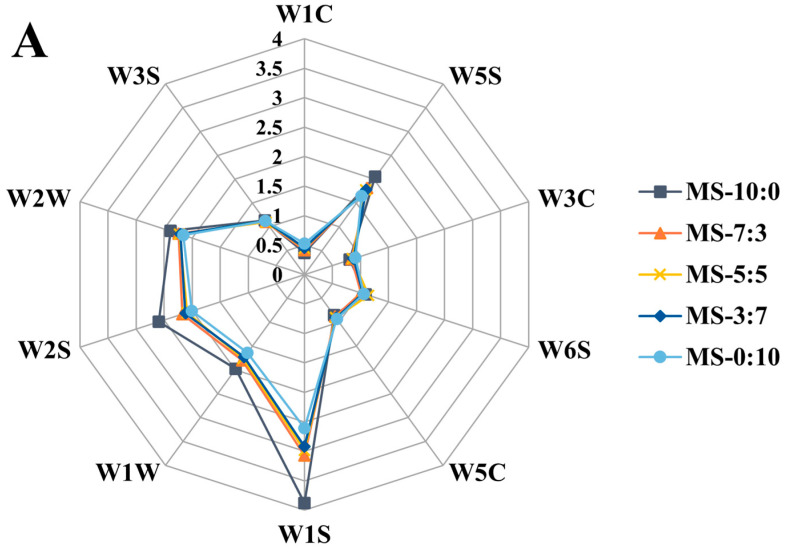

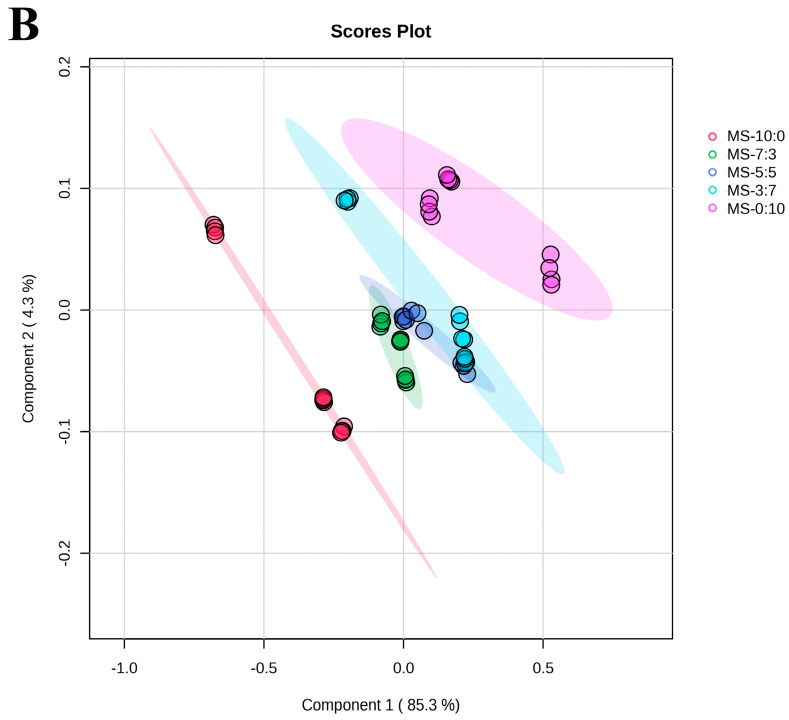
Odor radar map (**A**) and Partial Least Squares Discriminant Analysis (PLS-DA) plot (**B**) under different blend ratios of crucian carp soup.

There were few differences (*p* < 0.05) in the response intensities of the five groups of samples to sensors W3S, W5C, and W6S, indicating that there was a tiny difference between the content of alkane and hydride of the volatile components within the five crucian carp soup groups (Figure 4A). W1S had the highest response value, followed by W2S, W2W, W5S, and W1W, showing that crucian carp soup contains more methane. With the increase in RS addition, there was a significant difference (*p* < 0.05) in sensors W1C and W3C between the different blends of crucian carp soup, explaining why the fish residues differed more in aromatic components, ammonia, and aromatic amines after comminuting and high-pressure cooking.

In order to achieve a more effective and clear clustering analysis, the supervised PLS-DA model was further applied to distinguish the odor characteristics of different blends of crucian carp soup. In Figure 4B, R^2^ and Q^2^ are between 0.5 and 1, which indicates that the model has good predictive power. Among them, MS-10:0 and MS-0:10 are the farthest away, which indicates that the two groups of fish soup samples have the greatest difference in odor; MS-3:7 is near MS-0:10; and MS-5:5 is more similar to MS-7:3. In addition, MS-7:3 is closer to MS-10:0, indicating that the smell is more similar. The differences for specific flavor components in the different samples were not apparent. Therefore, GC-MS was used further to investigate the volatile components of crucian carp soup.

### 3.8. Volatile Flavor Component Analysis of Crucian Carp Soup

**Figure 5:**
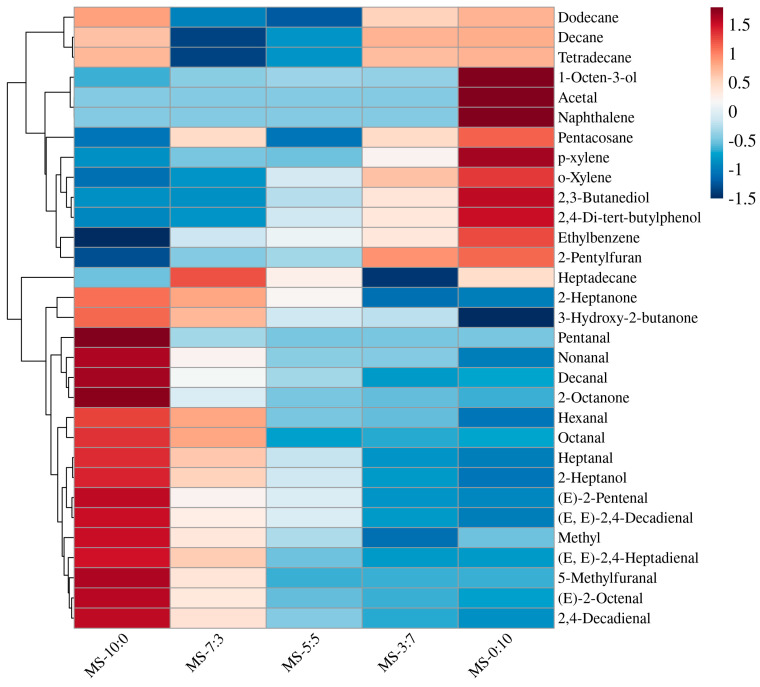
Heatmap visualization of the volatile flavor compounds under different blend ratios of crucian carp soup. The depth of the color represents the amount of volatile components contained, with redder colors indicating higher substance content, and bluer colors indicating lower substance content. The color legend explains the data value range corresponding to different colors.

There were many volatile substances in crucian carp soup; nevertheless, only some of them contributed to the flavor, which generally had high concentrations or expressed low thresholds [31]. As shown in Figure 5, a total of 31 volatile compounds were identified in the five sets of samples, including aldehydes, alcohols, ketones, esters, furans, and alkanes.

The key volatile odor components of crucian carp soup were mostly aldehydes, mainly produced by the oxidative degradation of polyunsaturated fatty acids, and had a fatty flavor [31]. Due to their low threshold value, they contributed to the formation of fish soup flavor. A total of 12, 12, 10, 9, and 9 aldehydes were detected from MS-10:0, MS-7:3, MS-5:5, MS-3:7, and MS-0:10, respectively. The aldehyde with the highest content of MS-0:10 was nonanal, and the other four groups had the highest content of hexanal. Most of these aldehydes have a fatty and grassy flavor. With the increase in the RS ratio, the content of aldehydes decreased significantly, which may be due to the further participation of some aldehydes in the Maillard reaction to generate volatile heterocyclic compounds [32].

Alcohols were mainly produced by the oxidative degradation of fats or the reduction of carbonyl compounds. Most alcohol thresholds are high and contribute rarely to the overall flavor of crucian carp soup [33]. In this study, 1-octene-3-ol was identified. It has mushroom, earthy, and oily flavors and is one of the characteristic flavors commonly found in fish soup. MS-0:10 has been detected at 2.75 µg/kg of 1-octene-3-ol, providing a distinctive aroma while enhancing the earthiness of the fish soup, which was consistent with sensory findings.

Ketones were mainly derived from the oxidation of unsaturated fatty acids or the thermal degradation of amino acids. Five groups of samples were detected to find the content of 2-heptanone, 3-hydroxy-2-butanone, and 2-octanone. These ketones generally presented fruity, creamy, and floral flavors, contributing a unique flavor to fish soup. With the expansion of the RS ratio, ketones content decreased gradually, which partly explained why MS-0:10 had the lowest sensory score.

2-Pentylfuran was also found in five groups of samples, with a low threshold value. Wall et al. [34] reported that linoleic acid and other n-6 fatty acids could generate 2-pentylfuran and have a plant aroma. Therefore, the heterocyclic compound provided a pleasant odor to crucian carp soup with the increased amount of RS.

The amount of volatile flavor compounds does not directly indicate a contribution to the overall flavor, thereby warranting an additional screening parameter (i.e., the threshold value) [35]. The ROAV of other volatile flavor compounds was further calculated to analyze the key flavor components of each sample and to describe the odor characteristics of important flavor compounds. A higher ROAV value indicates a greater contribution of that component to the overall flavor. Generally, when ROAV ≥ 1, the substance is considered the key flavor component in the sample. Volatile compounds with ROAV between 0.1 and 1 have an auxiliary function in the overall flavor presentation [31,35]. The results are shown in Table 2. Analysis of the ROAV results of crucian carp soup uncovered nine important odorants with ROAV > 0.1 and two key odorants with ROAV > 1. Table 2 shows that the main flavor components in the samples were changed with the increasing RS ratio. Overall, aldehydes were the most important flavor compounds in the crucian carp soup, with the main body of the soup showing fatty, grassy, and fruity aromas. The aldehyde content significantly decreased and the characteristic odor diminished when the amount of RS was increased.

## 4. Conclusions

Comminution combined with pressure-conduction cooking effectively promoted the leaching of more nutrients from the fish residue. The RS showed a higher utilization rate for raw materials and a fourfold increase in calcium content compared with the OS treatment. Through microscopic observation, comminution could promote the formation of MNCPs in crucian carp soup; the MNCPs of MS-7:3 formed stable and regular spheres with much smaller particle sizes. As the RS ratio increased, the content of histidine and IMP showed a decreasing trend, accompanied by a decrease in bitterness and freshness for the crucian carp soup. The ROAV > 1 of nonanal and (E, E)-2, 4-decadienal had a significant influence on the overall flavor formation, which made the main body of crucian carp soup show fat, grass and fruit flavor. With the increase in RS addition, the aldehyde content decreased and the characteristic odor diminished. In the sensory evaluation analysis, the MS-7:3 Umami was significantly higher than the other comparison groups (*p* < 0.05). Taking into account the nutritional and flavor characteristics of the product, 7:3 appeared to be the best compounding ratio for crucian carp soup. However, the high protein and oil content of fish soup requires further optimization of stability control during flavor retention and storage. At the same time, the bioactive application of the micro-nanoparticles and their effect on the flavor formation mechanism in fish soup need to be further investigated. 

## Figures and Tables

**Table 1:** Comparison of different chemical compositions of OS and RS.

Chemical Compositions	OS	RS
Total Solids (%)	2.10 ± 0.06 ^b^	12.18 ± 0.22 ^a^
Soluble Proteins (mg/100 mL)	13.20 ± 0.09 ^b^	82.36 ± 0.32 ^a^
Total Lipids (g/100 mL)	0.34 ± 0.03 ^b^	1.50 ± 0.01 ^a^
Total Proteins (g/100 mL)	1.30 ± 0.02 ^b^	4.39 ± 0.06 ^a^
Total Sugars (mg/100 mL)	13.75 ± 0.25 ^b^	36.04 ± 0.81 ^a^
Calcium Content (mg/L)	115.26 ± 0.71 ^b^	532.98 ± 0.68 ^a^

Note: each value was expressed as means ± S.D. (*n* = 3). Means with various lowercases s in the same line were significantly different (*p* < 0.05).

**Table 2:** Relative odor activity values (ROAV) and characteristic odor of volatile components under different blend ratios of crucian carp soup.

Compounds	Threshold (µg/kg)	Odor Characteristics	ROAV				
			MS-10:0	MS-7:3	MS-5:5	MS-3:7	MS-0:10
Pentanal	12	Almond, Bitter, Malt, Oil, Pungent	0.03	<0.01	/	/	/
Hexanal	4.5	Apple, Fat, Fresh, Green, Oil	100	100	100	100	0.18
(E)-2-Pentenal	1500	/	<0.01	<0.01	<0.01	<0.01	<0.01
Acetal	1000	Creamy, Fruit, Pleasant	/	/	<0.01	<0.01	<0.01
Heptanal	3	Citrus, Fat, Green, Nut	0.27	0.27	0.45	0.39	0.11
Octanal	0.7	Citrus, Fat, Green, Oil, Pungent	1.12	1.09	0.85	0.96	0.27
Nonanal	1	Fat, Floral, Green, Lemon	2.90	2.45	4.03	4.21	100
(E)-2-Octenal	3	Dandelion, Fat, Fruit, Grass, Green	0.13	0.08	0.04	0.03	0.01
(E, E)-2,4-Heptadienal	10	Fatty, Grassy	0.02	0.01	<0.01	<0.01	/
5-Methylfuranal	0.5	Fatty, Nutty	0.14	0.07	<0.01	/	/
2,4-Decadienal	0.3	Coriander, Deep Fried, Fat, Oil	0.72	0.44	0.28	0.10	/
(E, E)-2,4-Decadienal	0.07	Coriander, Deep Fried, Fat, Oil	25.95	16.58	25.35	10.08	1.40
Decanal	2.6	Sweet, Floral, Waxy, Fried	0.29	0.27	0.49	0.47	0.14
2,3-Butanediol	100,000	Sweet	/	/	<0.01	<0.01	<0.01
1-Octen-3-ol	1	Mushroom, Oily, Earthy	0.22	0.31	0.63	0.65	0.42
2-Heptanone	140	Citrus, Fried, Mushroom, Oil	<0.01	<0.01	<0.01	<0.01	<0.01
3-Hydroxy-2-butanone	800	Butter, Creamy, Green Pepper	<0.01	<0.01	<0.01	<0.01	<0.01
2-Octanone	50	Fat, Fragrant, Mold	<0.01	<0.01	<0.01	<0.01	<0.01
p-Xylene	450.23	/	<0.01	<0.01	<0.01	<0.01	/
o-Xylene	68.6	/	<0.01	<0.01	<0.01	<0.01	/
Ethylbenzene	2205.25	Fruity, Floral	<0.01	<0.01	<0.01	<0.01	<0.01
Naphthalene	60	Camphor	/	/	/	/	<0.01
Tetradecane	1000	/	<0.01	<0.01	<0.01	<0.01	<0.01
2-Pentylfuran	5.8	Butter, Floral, Fruit, Green Bean	0.04	0.13	0.28	0.54	0.17
2,4-Di-tert-butylphenol	200	Phenolic Flavor	<0.01	<0.01	<0.01	<0.01	<0.01

Note: (/)—Not identified.

## Data Availability

The original contributions presented in the study are included in the article, further inquiries can be directed to the corresponding author.

## Supplementary Materials

The following supporting information can be downloaded at: https://www.mdpi.com/article/10.3390/foods13050800/s1. Table S1. Scoring standard for basic attributes of crucian carp soup sensory evaluation; Table S2. Name and performance description of electronic nose sensor; Table S3. The mean intensity values of six attributes for crucian carp soup in descriptive sensory evaluation; Table S4. Free amino acids composition and contents under different blend ratios of crucian carp soup (wet basis).
