# Supplementary material for: Nutrient Composition and Flavor Profile of Crucian Carp Soup Utilizing Fish Residues through Comminution and Pressure-Conduction Treatment
Source: Foods. 2024 Mar 5;13(5):800. doi: 10.3390/foods13050800 (PMC10930992; doi:10.3390/foods13050800)
Supplement: Supplementary file 1 [file foods-13-00800-s001.zip › foods-2867160-supplementary.pdf]

Table S1. Scoring standard for basic attributes of crucian carp soup sensory evaluation

Sample property	0 points	3.5 points	7 points
Color	Milky white	Light yellow or light gray	Dark gray
Homogeneity	More oil precipitation, the system has obvious stratification, poor stability	Less grease precipitation, partially layered system, more stable	No obvious grease precipitation, the system is uniform and stable
Fishy odor	Sniffing the sample, the sample has a distinct fishy odor	Sniffing the sample, the sample has a weak fishy odor	Sniffing the sample, the sample has no fishy odor
Umami	Sniffing the sample, the sample has no fish flavor	Sniffing the sample, the sample has a weak fish flavor	Sniffing the sample, the sample has a distinct fish flavor
Taste	Tasting the sample, the sample has a distinct fishy flavor	Tasting the sample, the sample has a weak fishy flavor	Tasting the sample, the sample's fresh aroma is obvious
Texture	Coarse and grainy mouthfeel	More delicate mouthfeel, less grainy	Delicate mouthfeel, weak graininess

Table S2. Name and performance description of electronic nose sensor

Number	Name	Performance description
1	W1C	Sensitive to aromatic compounds
2	W5S	Sensitive to nitrogen oxides
3	W3C	Sensitive to amines and aromatic compounds
4	W6S	Selective for hydrides
5	W5C	Sensitive to alkanes and aromatic compounds
6	W1S	Sensitive to methyl groups
7	W1W	Sensitive to sulfide compounds
8	W2S	Sensitive to alcohols, aldehydes, and ketones
9	W2W	Sensitive to aromatic components and sulfides
10	W3S	Sensitive to alkanes and fatty compounds

Table S3. The mean intensity values of six attributes for crucian carp soup in descriptive sensory evaluation

Samples	Mean score					
	Color	Homogeneity	Fishy odor	Umami	Taste	Texture
MS-10:0	5.07±1.58^{a}	4.67±2.19^{a}	4.93±1.91^{a}	3.53±1.81^{b}	5.20±1.42^{a}	5.93±1.28^{a}
MS-7:3	3.20±1.27^{b}	3.87±1.41^{b}	4.53±1.77^{ab}	4.00±1.36^{a}	4.20±0.86^{ab}	4.67±1.23^{b}
MS-5:5	2.87±1.36^{bc}	3.53±1.36^{b}	3.73±1.44^{b}	3.73±1.44^{ab}	4.47±1.69^{ab}	4.27±1.39^{b}
MS-3:7	2.33±1.88^{bc}	3.93±1.75^{b}	4.60±1.35^{ab}	3.27±1.34^{b}	3.80±1.01^{b}	4.27±1.34^{b}
MS-0:10	1.87±1.25^{c}	3.67±2.09^{b}	3.87±2.13^{ab}	3.53±1.60^{b}	3.60±1.60^{b}	4.13±1.69^{b}

Note. Each value is expressed as means ± S.D. (n=3). Means with different letters in the same column are significantly different (p<0.05).

Table S4. Free amino acids composition and contents under different blend ratios of crucian carp soup (wet basis)

Free amino acids	Taste property	Contents /(mg/100mL)				
		MS-10:0	MS-7:3	MS-5:5	MS-3:7	MS-0:10
Essential amino acids						
Threonine	Sweet（+）	1.57±0.001[a]	1.20±0.001[b]	1.07±0.001[bc]	0.97±0.001[c]	0.47±0.001[d]
Valine	Bitter（-）	1.77±0.002[a]	1.43±0.001[b]	1.33±0.001[b]	1.03±0.002[c]	0.63±0.001[d]
Methionine	Sweet（+）	4.47±0.001[a]	4.83±0.004[a]	2.40±0.000[b]	2.37±0.002[b]	2.43±0.001[b]
Isoleucine	Bitter（-）	0.83±0.001[a]	0.67±0.001[a]	0.67±0.001[a]	0.43±0.001[b]	0.30±0.001[b]
Leucine	Bitter（-）	1.77±0.004[a]	1.40±0.003[ab]	1.43±0.002[ab]	1.10±0.001[b]	0.63±0.001[c]
Phenylalanine	Bitter（-）	1.50±0.002[a]	1.13±0.004[ab]	0.97±0.003[b]	0.83±0.003[bc]	0.43±0.001[c]
Lysine	Sweet（+）	4.63±0.003[a]	3.13±0.002[bc]	3.40±0.005[b]	2.57±0.003[c]	1.90±0.001[d]
Histidine	Bitter（-）	55.40±0.00[a]	45.60±0.007[b]	36.70±0.002[c]	30.33±0.002[d]	18.40±0.006[e]
Non-essential amino acids						
Serine	Sweet（+）	1.50±0.000[a]	1.23±0.001[b]	1.03±0.001[c]	0.90±0.000[c]	0.53±0.001[d]
Glutamic acid	Fresh（+）	2.03±0.002[a]	1.53±0.001[b]	1.43±0.001[bc]	1.23±0.001[c]	0.70±0.001[d]
Glycine	Sweet/ Fresh（+）	20.63±0.00[a]	16.27±0.003[b]	13.83±0.002[c]	11.37±0.001[d]	6.53±0.001[e]
Alanine	Sweet/ Fresh（+）	8.80±0.002[a]	6.83±0.003[b]	6.03±0.001[c]	4.93±0.001[d]	2.67±0.001[e]

Tyrosine	Bitter（-）	1.67±0.004[a]	1.23±0.004[ab]	1.10±0.002[bc]	0.93±0.001[bc]	0.60±0.001[c]
Total amino acids		106.53±0.005[a]	86.43±0.007[b]	71.42±0.001[c]	59.02±0.008[d]	36.26±0.008[e]

Note. +: indicates a pleasant, fresh or sweet taste; -: indicates a bad taste, bitter or sulphuric taste. Different letters in the same row indicate significant differences between samples ($p<0.05$).
